# Dose–Response Associations Between Screen-Based Digital Media Use and Anxiety in Children and Adolescents: A Systematic Review and Three-Level Meta-Analysis

**DOI:** 10.3390/bs16101774

**Published:** 2026-09-28

**Authors:** Wenxuan Lu, Shusheng Shi, Jiayu Chen

**Affiliations:** School of Physical Education, Nanjing Normal University, Nanjing 210023, China; luwx1023@163.com (W.L.);

**Keywords:** screen time, anxiety, adolescents, children, digital media, meta-analysis, dose–response, restricted cubic spline

## Abstract

Screen-based digital media use is widespread in childhood and adolescence, but its association with anxiety and dose–response relationship remain uncertain. We systematically reviewed observational studies of participants aged 6–19 years reporting time- or frequency-based screen exposure and anxiety-specific outcomes. PubMed/MEDLINE, Web of Science Core Collection, Scopus, and CNKI were searched through September 2026. Study quality was assessed using the AHRQ checklist or Newcastle–Ottawa Scale. Eligible estimates were synthesized using three-level random-effects meta-analysis and restricted cubic splines. Fifty-seven reports were retained. The primary adjusted dataset included 79 odds-ratio effects from 17 independent studies (pooled OR 1.40, 95% CI 1.26–1.54; I^2^ = 51.7%). Screen type significantly moderated the association after Holm correction. The dose–response analysis included seven independent studies; the linear component was significant (*p* = 0.039), whereas nonlinearity was not supported (*p* = 0.132; wild-cluster *p* = 0.163). Evidence was predominantly observational and cross-sectional, and dose–response data were limited. Greater screen exposure was associated with higher odds of anxiety, supporting a graded pattern without a discrete threshold. Because the evidence was predominantly observational and cross-sectional, this exposure–response gradient should be interpreted as an association rather than evidence that greater screen use causes anxiety; reverse and reciprocal pathways remain possible. This review was not registered and was funded by two Jiangsu Provincial Education Science Planning projects.

## 1. Introduction

Anxiety is one of the most common mental health problems during childhood and adolescence and can interfere with school participation, social functioning, sleep, and longer-term well-being. The World Health Organization estimates that approximately one in seven adolescents aged 10–19 years experiences a mental disorder and identifies anxiety disorders among the leading causes of illness and disability in this age group; estimated anxiety disorder prevalence rises from 4.1% among 10–14-year-olds to 5.3% among 15–19-year-olds (World Health Organization, 2025). Adolescence is also a period of rapid neurobiological, cognitive, and social development, during which sensitivity to peer evaluation, social feedback, uncertainty, and stress is heightened. These developmental features make anxiety particularly relevant when considering environments that are socially interactive, continuously available, and capable of extending peer experiences beyond face-to-face settings (Orben et al., 2024).

Screen-based digital media have become embedded in the everyday lives of young people. Smartphones, social media platforms, online video, gaming, computers, and other connected devices now serve educational, recreational, and interpersonal functions, making screen exposure difficult to conceptualize as a single homogeneous behavior. In a 2024 probability-based survey of U.S. adolescents aged 13–17 years, 95% had access to a smartphone, and 46% reported being online almost constantly (Faverio & Sidoti, 2024). At the same time, public health guidance has become more nuanced. The Canadian 24-Hour Movement Guidelines recommend no more than 2 h/day of recreational screen time for children and youth aged 5–17 years (Tremblay et al., 2016), whereas current American Academy of Pediatrics guidance emphasizes that evidence is insufficient to define a single universally safe time limit for all children and adolescents and recommends considering the quality and context of digital media use in addition to duration (Munzer et al., 2026). These differing approaches highlight the need to characterize the association across the full exposure range rather than assuming that one cutoff point necessarily represents a biological threshold.

Sedentary behavior, screen-based sedentary behavior, screen time, and digital media use are related but non-equivalent constructs. For the purposes of this review, sedentary behavior refers to the broader behavioral domain, whereas screen-based sedentary behavior is the subset of behaviors involving devices with screens. Screen time refers to the duration or frequency of viewing or interacting with screen-based devices, while digital media use may additionally vary by platform, content, interactivity, purpose, and social context. The target exposure synthesized here was screen-based digital media use quantified by actual duration or frequency; general sedentary behavior was not synthesized as the target exposure, and problematic or addictive digital media use constructs without an identifiable measure of actual use were excluded. Accordingly, the broader sedentary behavior literature is used as contextual rather than directly equivalent evidence. Displacement pathways involving sleep, physical activity, or face-to-face activity are distinguished from media-specific pathways such as social comparison, online feedback, cybervictimization, reassurance seeking, and exposure to socially salient content.

Previous syntheses generally support a positive association between sedentary behavior or digital media exposure and anxiety, but the magnitude and interpretation of this association remain uncertain. An earlier meta-analysis of sedentary behavior reported a small positive overall association with anxiety (weighted r = 0.093), while emphasizing the need for longitudinal evidence and better differentiation of sedentary behavior types (Stanczykiewicz et al., 2019). More recent anxiety-specific work has focused heavily on social media. Kerr et al. (2025) included 32 studies and found that 56.3% reported positive social media–anxiety associations; positive findings were particularly common for screen time measures. Fassi et al. (2024) synthesized 143 studies of social media use and internalizing symptoms and found small positive meta-correlations for time spent on social media, while also documenting substantial methodological heterogeneity. During the COVID-19 period, Yoshizawa et al. (2026) reported a pooled correlation of r = 0.157 between screen time and anxiety across pediatric studies. Collectively, these reviews establish that an association is plausible, but they do not provide a comprehensive estimate focused specifically on actual time-based screen exposure and anxiety across multiple screen types while simultaneously addressing dependence among multiple effects from the same study.

Important gaps therefore remain. First, many previous reviews combined anxiety with depression or broader internalizing outcomes, or combined actual duration with problematic use/addiction constructs that represent conceptually different exposures (Ahmed et al., 2024; Saleem et al., 2024). Second, individual studies often report multiple screen types, exposure categories, sex strata, or anxiety outcomes from the same sample; conventional meta-analytic approaches can discard information or underestimate uncertainty when these effects are treated as independent. Third, the nature of the time–anxiety association is poorly defined. A recent multilevel meta-analysis of adolescent screen time and depression reported a nonlinear relationship and an overall risk threshold near 2 h/day (Guan et al., 2026), but whether a comparable threshold exists for anxiety is unknown. This distinction matters because anxiety may be influenced not only by sedentary duration, but also by social-evaluative exposure, anticipatory arousal, reassurance seeking, fear of missing out, and sleep disruption, potentially producing a different dose–response pattern.

Accordingly, we conducted a systematic review and three-level meta-analysis to synthesize the association between screen-based digital media use and anxiety among children and adolescents. The specific aims were to (1) estimate the overall association between greater screen-based digital media exposure and anxiety; (2) examine whether the association varies according to screen type, study design, region, outcome ascertainment, and, where data permit, sex; and (3) characterize the dose–response relationship between daily screen exposure and anxiety using restricted cubic spline models, including formal tests of nonlinearity and sensitivity analyses for dose assignment. By distinguishing actual time-based exposure from problematic use constructs and explicitly modeling dependent effect sizes, this study aims to provide a more precise characterization of how the quantity and type of screen use relate to anxiety during childhood and adolescence. Throughout this review, “dose–response” describes the nature of the observed exposure–outcome association across increasing exposure levels and does not, by itself, establish causal direction.

## 2. Materials and Methods

### 2.1. Reporting Framework and Protocol

This systematic review and meta-analysis was conducted and reported in accordance with the Preferred Reporting Items for Systematic Reviews and Meta-Analyses (PRISMA) 2020 statement (Page et al., 2021). A completed PRISMA 2020 checklist is provided as separate reporting material, and the study selection process is presented in Figure 1. The review focused on time-based screen and digital media exposures and anxiety outcomes in children and adolescents. The primary quantitative synthesis used a three-level meta-analytic structure to retain multiple eligible effect sizes from the same study while accounting for within-study dependence. Reports arising from overlapping cohorts were identified during the full-text review and were not treated as independent primary studies when they represented the same underlying sample. The review was not prospectively registered. No formal review protocol was prepared; accordingly, registration- or protocol-based amendments were not applicable.

### 2.2. Information Sources and Search Strategy

PubMed/MEDLINE, Web of Science Core Collection, Scopus, and China National Knowledge Infrastructure (CNKI) were searched in September 2026. The search combined terms for children/adolescents, screen-based digital media exposure, and anxiety. Dose, duration, or hour terms were not required in the main search because dose–response eligibility was determined at full-text review. The final database yields recorded in the analysis workbook were 3837 records from PubMed/MEDLINE, 7083 from Web of Science, 8274 from Scopus, and 0 from the final CNKI advanced search. Full reproducible search strings are provided in the supplementary search strategy workbook.

### 2.3. Eligibility Criteria

Eligibility was defined according to the PICOS framework (Participants, Intervention/Exposure, Comparator, Outcome, and Study design).

Participants (P). Studies were eligible if they included children or adolescents with a mean age between 6 and 19 years at the time of screen exposure assessment. Studies involving broader age ranges were considered only when data for the eligible child or adolescent subgroup could be extracted separately.

Intervention/Exposure (I/E). The exposure of interest was time-based screen-based digital media use. Eligible exposures included total screen time, television or video viewing, computer or Internet use, smartphone or mobile phone use, social media or screen-based communication, and video gaming. Self-reported, parent-reported, and objectively measured screen exposure were eligible. Studies assessing only problematic or addictive digital media use using psychometric scales, without an identifiable measure of actual use duration or frequency, were excluded. General sedentary behavior without a screen-based digital media exposure measure was not treated as the target exposure.

Comparator (C). Eligible studies compared higher levels of screen-based digital media exposure with a lower or reference exposure category or reported associations per unit increase in screen exposure. For dose–response analyses, the lowest reported exposure category was preferentially used as the reference group when available.

Outcome (O). The primary outcome was anxiety, including anxiety symptoms, elevated or clinically relevant anxiety, and diagnosed anxiety disorders. Generalized anxiety and social anxiety were eligible when reported as distinct anxiety outcomes. Anxiety could be assessed using validated symptom scales, clinical interviews, diagnostic criteria, or health-record diagnoses. Studies reporting only composite outcomes such as internalizing problems, psychological distress, or combined anxiety/depression scores were excluded, unless an anxiety-specific effect estimate could be extracted separately.

Study design (S). Original observational studies were eligible and were classified for analysis as either cross-sectional or longitudinal. Prospective cohort and repeated-measures analyses were treated as longitudinal when the extracted association incorporated temporal ordering between screen exposure and a subsequent anxiety assessment; estimates based on exposure and anxiety measured at the same assessment occasion were treated as cross-sectional, even when they arose from a longitudinal parent cohort. Studies were required to report an effect estimate for the association between screen-based digital media use and anxiety (e.g., OR, RR, PR, HR, correlation coefficient, or regression coefficient) or provide sufficient data from which an effect estimate and its variance could be calculated.

Studies were excluded if they (1) were reviews, meta-analyses, conference abstracts, protocols, editorials, corrections without original data, preprints, or other non-peer-reviewed reports; (2) included an ineligible population without separately extractable data for children or adolescents; (3) assessed only problematic/addictive digital media use without an actual time- or frequency-based exposure measure; (4) did not report an anxiety-specific outcome; (5) did not provide sufficient quantitative information for effect-size extraction or calculation; (6) examined anxiety as the exposure and screen use as the outcome without reporting the association in the direction relevant to the present review; or (7) reported data from a population that substantially overlapped with another included report, in which case the most informative and methodologically appropriate report was retained for the primary analysis, and overlapping reports were reserved for sensitivity analyses when informative.

### 2.4. Study Selection and Data Extraction

After database export, exact DOI/title matching was used for deduplication. Title/abstract screening and the initial full-text assessment were conducted by W.L. according to the prespecified eligibility criteria; all reports retained in the review were subsequently independently verified by J.C. against the original reports. No automated or machine learning study selection system made eligibility decisions. Data were extracted at the effect size level by W.L. and J.C. and independently cross-checked against the source articles before inclusion in the qualitative and quantitative syntheses. Variables sought included study and cohort identifiers, publication year, country/region, study design, sample size and age, exposure definition and measurement, screen type, exposure category or dose, anxiety outcome and instrument, effect metric, comparison contrast, adjustment status and covariates, effect estimate, confidence interval or other variance information, sex stratum and assessment occasion where available, and information required for dose assignment. Adjusted estimates were preferred when available. Multiple eligible effects from a single report were retained for the three-level model. Original effect metrics were preserved during extraction; ORs, RRs, and PRs were not relabeled as one another. Unclear or missing information was not imputed unless a prespecified and defensible transformation could be applied from reported data. Any discrepancies identified during verification were resolved through discussion and re-examination of the original report until consensus was reached.

### 2.5. Data Harmonization and Effect Size Conversion

Effect estimates were harmonized before quantitative synthesis. Whenever multiple estimates were available, the most fully adjusted estimate was preferentially extracted. Crude and adjusted estimates were not combined in the primary analysis. Effect directions were standardized so that values greater than 1.00 consistently represented a higher likelihood of anxiety associated with greater screen-based digital media exposure. When an original study reported the comparison in the opposite direction, the effect estimate and confidence limits were inverted accordingly.

Odds ratios (ORs) were the primary effect measure for the main three-level meta-analysis. ORs were transformed to the natural logarithmic scale before analysis. This transformation was used because the sampling distribution of an OR is generally more nearly normal and its confidence interval more symmetric on the log scale, while multiplicative associations become additive for inverse variance meta-analytic modeling. Pooled estimates and confidence intervals were exponentiated back to the OR scale for interpretation. For estimates reported with a 95% confidence interval (CI), the standard error (SE) of the log OR was calculated as SE = [ln(upper CI) − ln(lower CI)]/(2 × 1.96). For 99% CIs, the corresponding critical value of 2.576 was used. When sufficient cell count data were available, crude ORs were reconstructed directly from 2 × 2 contingency tables and were clearly distinguished from multivariable-adjusted estimates (Borenstein et al., 2009).

Risk ratios (RRs), prevalence ratios (PRs), and hazard ratios (HRs) were not automatically treated as equivalent to ORs because these measures estimate different quantities, particularly when the outcome is not rare. Conversion of an RR to an OR was considered only when the baseline risk in the reference group was available and the transformation was defensible, using OR = RR(1 − P0)/(1 − RR × P0), where P0 denotes the outcome risk in the non-exposed/reference group (Zhang & Yu, 1998). Otherwise, non-OR measures were retained in their original metric and analyzed separately or in sensitivity analyses.

Correlation coefficients were transformed using Fisher’s z transformation, z = 0.5 × ln[(1 + r)/(1 − r)], with SE = 1/√(n − 3), when synthesis on the correlation scale was required (Borenstein et al., 2009). Regression coefficients derived from continuous anxiety scales were retained as continuous outcome effects and were not mechanically converted to ORs. Exponentiation of a regression coefficient was interpreted as an OR only when the coefficient originated from an appropriate logistic regression model.

For dose–response analyses, screen exposure was standardized to hours per day whenever a valid conversion was possible. Exposure categories defined by closed intervals were assigned the reported mean or median exposure; when these were unavailable, the midpoint of the category boundaries was used. Open-ended upper categories were assigned values according to a prespecified extrapolation rule based on the width of the adjacent category, with alternative assignments examined in sensitivity analyses. Dose–response estimation from summarized categorical data followed established generalized least-squares principles, and nonlinear trends were modeled using restricted cubic splines (Greenland & Longnecker, 1992; Orsini et al., 2012). Restricted cubic splines are piecewise cubic functions joined smoothly at prespecified knots and constrained to be linear beyond the boundary knots, limiting unstable curvature in the sparsely observed tails while retaining flexibility within the observed exposure range. Measures expressed specifically as hours per school day or other non-equivalent time units were not included in the primary hours-per-day model unless a defensible conversion was available.

When multiple eligible effect estimates were reported within the same study—for example, by sex, screen type, anxiety subtype, exposure category, or assessment occasion—eligible effects were retained rather than arbitrarily selecting a single estimate. Their statistical dependence was addressed in the three-level meta-analytic framework and, where appropriate, with study cluster robust inference (Assink & Wibbelink, 2016; Hedges et al., 2010; Tipton, 2015; Tipton & Pustejovsky, 2015).

### 2.6. Study Quality Assessment

Methodological quality was assessed with design-appropriate instruments. Longitudinal studies were evaluated using the Newcastle–Ottawa Scale (NOS), which assesses selection, comparability, and outcome domains; scores of 0–3, 4–6, and ≥7 were classified as low, moderate, and high quality, respectively (Stang, 2010). Cross-sectional studies were evaluated using the 11-item checklist recommended by the Agency for Healthcare Research and Quality (AHRQ); items were scored 1 for “yes” and 0 for “no” or “unclear”, yielding total scores of 0–11, with 0–3, 4–7, and 8–11 classified as low, moderate, and high quality, respectively (Rostom et al., 2004). The initial quality appraisal was conducted by W.L. and subsequently independently verified in full by J.C. against the original reports. Any discrepancies in item-level judgments or overall ratings were resolved through discussion and re-examination of the source study until consensus was reached. The final consensus assessments were used in all descriptive summaries, sensitivity analyses, and graphical presentations.

### 2.7. Statistical Analyses

The primary analysis used adjusted odds ratios and was fitted with a three-level random effects model. At Level 1, each effect contributed its sampling variance (v_i_ = SE_i_^2^), reflecting uncertainty associated with estimation of that effect and treated as an input to the model. Level 2 represented residual heterogeneity among multiple eligible effects nested within the same study, whereas Level 3 represented heterogeneity between independent studies (Assink & Wibbelink, 2016). Models were estimated in R using the metafor framework (Viechtbauer, 2010). Heterogeneity was summarized using variance components and I^2^-type measures (Higgins & Thompson, 2002). The primary model was based on the fully adjusted OR dataset; crude reconstructed estimates and non-OR metrics were reserved for sensitivity or secondary analyses.

Dose–response associations were modeled using restricted cubic splines based on the harmonized hours-per-day dataset (Greenland & Longnecker, 1992; Orsini et al., 2012). The primary analysis used three knots at the 10th, 50th, and 90th percentiles of assigned doses (0.25, 1.50, and 4.50 h/day) and tested linear and nonlinear spline components separately. Open-ended categories were assigned using a prespecified adjacent-width rule, with alternative assignments examined in sensitivity analyses. The independent study was used as the clustering unit for study cluster sandwich inference. Given that only seven independent study clusters contributed to the primary spline model, inference was additionally checked by exact enumeration of all 2^7^ = 128 study-level Rademacher sign patterns under the restricted linear null (Hedges et al., 2010; Tipton, 2015; Tipton & Pustejovsky, 2015; Cameron et al., 2008). A threshold was not declared unless the nonlinear component was statistically supported and the apparent turning point was stable across sensitivity analyses.

Prespecified moderator analyses examined screen type, region, study design, and anxiety outcome ascertainment. For moderator meta-regression, the independent study was treated as the clustering unit. Cluster-robust standard errors were obtained from a study-level sandwich covariance estimator applied to the fitted three-level model, with CR1-type finite-sample scaling, [G/(G − 1)] × [(n − 1)/(n − p)], where G is the number of independent study clusters, n is the number of effect estimates, and p is the number of model coefficients. Omnibus moderator tests used F statistics with numerator degrees of freedom equal to the number of moderator coefficients tested and denominator degrees of freedom G − p. Holm adjustment was retained as the primary multiplicity-control procedure for the four prespecified omnibus tests; as a sensitivity analysis, the same raw *p* values were additionally adjusted using the Benjamini–Hochberg false discovery rate procedure (Benjamini & Hochberg, 1995). Sex-stratified results were summarized descriptively because only three independent studies provided explicit male/female strata. Small-study effects were explored using funnel plots and regression-based asymmetry diagnostics, interpreted cautiously because multiple effects were nested within studies; the Egger framework was treated as an exploratory diagnostic rather than definitive evidence for or against publication bias (Egger et al., 1997). Statistical evidence was interpreted using two-sided tests with *p* < 0.05, while sparse subgroup analyses were treated as exploratory.

Eligibility for each synthesis was determined using the prespecified effect metric and dose recoverability rules. The broad primary synthesis included fully adjusted OR-family estimates representing greater screen exposure in relation to anxiety presence or worsening. Most effects were study-defined categorical higher-versus-lower contrasts, while a small number were continuous/per-unit, transition/change, or mixed movement guideline contrasts. These contrasts were not assumed to represent an identical quantitative increase in exposure; accordingly, the pooled OR was interpreted as an average association across study-defined contrasts rather than as the effect associated with a fixed increment in screen time. To evaluate the influence of contrast-scale heterogeneity, an additional sensitivity analysis was restricted to conventional categorical higher-versus-lower comparisons, excluding per-unit, transition/change, and mixed movement guideline contrasts. Crude/reconstructed ORs and non-OR metrics were reserved for sensitivity or secondary analyses. The primary dose–response synthesis required recoverable quantitative exposure levels that could be expressed in hours/day, together with anxiety-specific effect estimates. Study characteristics and quantitative findings were summarized in tables and displayed using forest plots, restricted-cubic-spline curves, moderator plots, leave-one-study-out plots, and an exploratory funnel plot. Robustness was examined through broader OR-family, strict-contrast, categorical-only, high-quality-only, leave-one-study-out, alternative dose assignment and knot placement, and dose–response leave-one-study-out analyses.

No formal certainty-of-evidence framework (e.g., GRADE) was applied; therefore, no overall certainty rating was assigned. Robustness was instead characterized using study quality assessments, heterogeneity estimates, prediction intervals, sensitivity analyses, influence analyses, and small study effect diagnostics.

Generative artificial intelligence (ChatGPT, GPT-5.6 Sol; OpenAI) was used as an assistive tool for organizing review materials, supporting structured data extraction and analytical workflow preparation, assisting with manuscript revision, and preparing tabular outputs. It did not replace author judgment or make final eligibility decisions autonomously. All eligibility decisions, extracted data, statistical inputs and outputs, interpretations, and final manuscript content were reviewed and verified by the authors against the source materials and analysis records.

## 3. Results

### 3.1. Study Selection

The four-database search identified 19,194 records. After removal of 7416 duplicates, 11,778 unique records underwent title/abstract screening, of which 11,680 were excluded. Ninety-eight reports were sought for full-text retrieval; twenty-three could not be retrieved, and seventy-five were assessed for eligibility. Eighteen reports were excluded after full-text assessment: five because the eligible child/adolescent population could not be separated, six because anxiety was not reported as a separate eligible outcome, three because of ineligible publication type/design, three because no isolated variance-ready screen-time–anxiety association could be extracted, and one because the target exposure/outcome construct was ineligible. Fifty-seven reports were retained for the systematic review and quantitative follow-up, comprising fifty-three primary follow-up reports and four sensitivity/overlap reports. Report-level counts and summarized exclusion reasons are shown in Figure 1; citations and report-specific reasons for all full-text exclusions are provided in Supplementary Table S4. Cohort overlap handling was performed separately for quantitative analyses.

### 3.2. Study Quality

Among the 57 retained reports, 29 were rated high quality, 27 moderate quality, and 1 low quality. The characteristics, analytic roles, dose grades, and quality scores of the retained reports are summarized in Table 1 (Alsaigh et al., 2022; Anjum et al., 2022; Boers et al., 2020; Carlander et al., 2026; Chen et al., 2024; Choi et al., 2025; Dabravolskaj et al., 2023; Dai & Ouyang, 2026; Faryabi et al., 2024; Feng & Tan, 2026; Fortier et al., 2025; Francisquini et al., 2025; Frei et al., 2025; Frielingsdorf et al., 2025; Gunnell et al., 2016; Hamshari et al., 2024; Hrafnkelsdottir et al., 2018; Hua et al., 2025; Karakus et al., 2026; Kim et al., 2020; Kosola et al., 2024; Lema-Gómez et al., 2021; Leung & Torres, 2021; D. Li et al., 2024; S. H. Li et al., 2025; Liu et al., 2016; Mandic et al., 2024; Maras et al., 2015; Menezes et al., 2023, 2024; Mohd Saat et al., 2024; Mougharbel et al., 2023; Mundy et al., 2021; Nercessian & Salameh, 2025; Nguyen et al., 2022; Oberle et al., 2020; Oliva et al., 2021; Parida et al., 2025; Pedrini et al., 2024; Roberston et al., 2022; Sârbu et al., 2024; Selfhout et al., 2009; Sidiq et al., 2025; Smout et al., 2023; Thorisdottir et al., 2019; Uddin & Hasan, 2023; Wang et al., 2025; Wen et al., 2022; M. Xiang et al., 2022; Z. Xiang et al., 2022; Xu et al., 2026; Yalçınkaya et al., 2023; Yan et al., 2017; Yang et al., 2026; Zablotsky et al., 2025; Zetterqvist et al., 2026; Zimmerman et al., 2024).The mean AHRQ score among 45 cross-sectional reports was 7.11/11, and the mean NOS score among 12 longitudinal reports was 7.58/9. These values represent the final consensus ratings following independent verification by J.C. Restricting the primary quantitative synthesis to high-quality studies yielded a pooled OR of 1.36 (95% CI 1.18–1.56), consistent in direction and magnitude with the primary model. Item-level quality-assessment results are shown in Figure 2.

### 3.3. Overall Association Between Screen-Based Digital Media Use and Anxiety

The primary three-level meta-analysis included 79 adjusted OR-family effects from 17 independent studies. The main quantitative results are presented in Table 2. Higher screen-based digital media use was associated with greater odds of anxiety (pooled OR = 1.40, 95% CI 1.26–1.54). Total heterogeneity was moderate (I^2^ = 51.7%), with approximately 25.8% attributable to within-study heterogeneity and 25.9% to between-study heterogeneity. The 95% prediction interval was 0.91–2.14. The corresponding forest plot is shown in Figure 3; the complete effect-level display is provided in Figure S1.

Because the broad synthesis included several nonconventional exposure contrasts, an additional sensitivity analysis was restricted to conventional categorical higher-versus-lower comparisons. This analysis excluded seven per-unit, transition/change, or mixed movement guideline effects and retained 72 effects from 12 independent studies. The pooled association was essentially unchanged (OR = 1.40, 95% CI 1.24–1.58), indicating that the overall estimate was not materially driven by the noncategorical contrasts.

### 3.4. Dose–Response Association

The primary restricted cubic spline analysis included 55 non-reference effects representing 19 dose curves from 7 independent studies with exposure expressed in hours/day. The linear spline component was statistically significant (*p* = 0.0394), whereas the nonlinear component was not (*p* = 0.1316). Exact wild cluster bootstrap inference likewise did not support nonlinearity (*p* = 0.1628). Accordingly, the evidence was interpreted as a monotonic increasing dose–response association rather than a threshold or change-point relationship. The fitted dose–response curve is shown in Figure 4.

Relative to the model reference of 0 h/day, predicted odds ratios were 1.05 at 1 h/day, 1.13 at 2 h/day, 1.25 at 3 h/day, 1.41 at 4 h/day, and 1.60 at 5 h/day. Sensitivity analyses that incorporated h/school day exposures, altered open-ended category assumptions, or changed knot locations consistently failed to show robust nonlinearity.

### 3.5. Moderator Analyses

Screen type significantly moderated the association after Holm correction (adjusted *p* = 0.000051) and remained significant under Benjamini–Hochberg FDR adjustment (q = 0.000051). The pooled OR was 1.54 (95% CI 1.32–1.80) for total/mixed screen exposure, 1.33 (0.99–1.79) for social/internet/communication exposure, 1.16 (0.90–1.51) for gaming/interactive exposure, and 1.12 (0.75–1.69) for passive/television exposure. These subgroup estimates should be interpreted cautiously because several categories contained only three or four independent studies. Subgroup estimates are summarized in Figure 5.

Outcome ascertainment was also a statistically significant moderator with both Holm correction (adjusted *p* = 0.0483) and Benjamini–Hochberg FDR adjustment (q = 0.0322); symptom scale/cutoff studies showed a pooled OR of 1.46 (95% CI 1.28–1.66), compared with 1.20 (1.11–1.30) for diagnosis/condition outcomes. Region was sensitive to the multiplicity procedure: the raw *p* value was 0.0359, corresponding to Holm-corrected *p* = 0.0718 but Benjamini–Hochberg–adjusted q = 0.0479. Study design was not a significant moderator under either procedure (Holm-adjusted *p* = 0.1655; BH q = 0.1655). The regional finding was therefore treated as exploratory.

### 3.6. Influence and Small Study Effects

Leave-one-study-out analyses yielded pooled ORs ranging from approximately 1.36 to 1.42. No study exceeded the prespecified DFBETAS influence threshold of 2/√17 (0.485), indicating that the overall result was not driven by a single study. The largest absolute DFBETAS value was 0.447. The leave-one-study-out estimates are shown in Figure 6.

There was no consistent statistical evidence of small study effects. In the three-level meta-regression, the standard error slope test was non-significant (*p* = 0.838). The cluster-robust Egger intercept was also non-significant (*p* = 0.735), as was the study-balanced cluster-robust sensitivity analysis (*p* = 0.251). Because effect sizes were dependent and represented heterogeneous exposure contrasts, the funnel plot was treated as exploratory rather than as definitive evidence for or against publication bias. The exploratory funnel plot is shown in Figure 7. Because no formal certainty-of-evidence framework was applied, no GRADE-style certainty ratings are presented.

## 4. Discussion

This systematic review and meta-analysis synthesized the available evidence on time-based screen and digital media exposure in relation to anxiety among children and adolescents. The primary three-level model showed that greater screen exposure was associated with higher odds of anxiety (OR = 1.40, 95% CI 1.26–1.54), with moderate total heterogeneity (I^2^ = 51.7%). The dose–response analysis further indicated progressively higher observed odds of anxiety across increasing levels of daily screen exposure. However, unlike the nonlinear pattern reported for adolescent depression in the reference dose–response literature (Guan et al., 2026), the nonlinear spline component was not statistically supported in the present analysis (*p* = 0.132; exact wild cluster bootstrap *p* = 0.163). The most defensible interpretation is therefore a monotonic increasing association rather than a discrete threshold effect. Screen type and anxiety outcome ascertainment were significant with both Holm and Benjamini–Hochberg adjustment; regional moderation met the FDR criterion but not the more conservative Holm criterion, while study design did not show robust moderation. The overall association was stable in leave-one-study-out and high-quality-only analyses, and it was essentially unchanged when the synthesis was restricted to conventional categorical higher-versus-lower contrasts (OR = 1.40, 95% CI 1.24–1.58).

The direction of the overall association is consistent with previous syntheses, although direct numerical comparisons should be made cautiously because earlier studies used different exposure definitions and effect metrics. Meta-analytic and review evidence generally suggests small positive associations between sedentary or screen-based behaviors and anxiety or broader internalizing outcomes (Stanczykiewicz et al., 2019; Kerr et al., 2025; Fassi et al., 2024; Yoshizawa et al., 2026; Ahmed et al., 2024; Saleem et al., 2024; Tang et al., 2021; Stiglic & Viner, 2019; Zink et al., 2020; Zhang et al., 2022; Mougharbel & Goldfield, 2020). Our findings extend this literature in two ways. First, the exposure was deliberately restricted to actual time- or frequency-based digital media use rather than problematic use or addiction scores. Second, multiple eligible effects from the same study were retained within a three-level model instead of being discarded or treated as statistically independent. Thus, the broad pooled estimate should be understood as an average association across study-defined exposure contrasts and heterogeneous screen contexts, not as the OR associated with one fixed increase in hours/day or as evidence that all forms of digital engagement carry equivalent risk. Importantly, excluding the seven per-unit, transition/change, or mixed movement guideline effects produced an essentially identical categorical-only pooled estimate, supporting the robustness of the overall association to contrast-scale heterogeneity.

A central finding was the absence of robust statistical evidence for a nonlinear threshold. The restricted cubic spline predicted progressively higher odds of anxiety across the observed range: approximately 1.05 at 1 h/day, 1.13 at 2 h/day, 1.25 at 3 h/day, 1.41 at 4 h/day, and 1.60 at 5 h/day relative to the model reference of 0 h/day. These estimates describe a dose gradient and should not be interpreted as absolute probabilities or clinical cutoff points. In particular, the 2 h/day point should not be labeled an anxiety threshold merely because it resembles a commonly used recreational screen time recommendation. The Canadian 24-Hour Movement Guidelines retain a ≤ 2 h/day recreational screen time recommendation (Tremblay et al., 2016), whereas the American Academy of Pediatrics’ updated digital ecosystem policy emphasizes context, content, developmental needs, displacement of sleep and movement, and family-specific boundaries rather than a universal “safe” number of hours (Munzer et al., 2026). Our findings are more consistent with a graded-association interpretation than with a biologically sharp cutoff. They also differ from the nonlinear pattern reported by Guan et al. (2026) for adolescent depression, suggesting that the shape of screen time associations may differ across mental health outcomes and analytic datasets.

Screen type significantly moderated the association. Total or mixed screen exposure showed the largest pooled estimate (OR = 1.54), followed by social/internet/communication exposure (OR = 1.33), whereas gaming/interactive (OR = 1.16) and passive/television exposure (OR = 1.12) had smaller and less precise estimates. These differences should be interpreted cautiously because several screen-type subgroups contained only three or four independent studies. Nevertheless, the pattern supports the broader view that “screen time” is not a unitary exposure. Interactive and socially evaluative media can involve continuous feedback, social comparison, exclusion, cyberbullying, emotionally salient content, and pressure to remain connected, whereas passive viewing and gaming may involve different combinations of stimulation, social interaction, and displacement of offline activities (Orben et al., 2024; Saleem et al., 2024). At the same time, the relatively large estimate for total/mixed screen time cautions against assuming that only social media matters; aggregated screen exposure may also capture cumulative displacement of sleep, physical activity, in-person interaction, or other restorative behaviors.

Several pathways could plausibly contribute to the observed association. Sleep is one of the most consistently proposed mechanisms: prolonged or late-night digital media use can displace sleep, delay bedtime, increase cognitive or emotional arousal, and expose adolescents to alerts or socially salient interactions at times when disengagement is difficult. Social comparison and feedback-seeking may heighten self-evaluative concerns, while cyberbullying and negative online interactions can act as direct social stressors. Conversely, anxiety itself may increase screen use through avoidance of offline situations, reassurance seeking, passive browsing, or preference for digitally mediated interaction. This reciprocal possibility is important because the pooled estimate was derived largely from observational evidence. Reviews of longitudinal research similarly caution that directionality is difficult to establish and that associations are likely to vary across individuals and contexts (Ahmed et al., 2024; Saleem et al., 2024; Tang et al., 2021; Zetterqvist et al., 2026). Therefore, the present findings should not be interpreted as demonstrating that additional screen time directly causes anxiety in every child or adolescent.

Study design did not significantly moderate the association after adjustment for multiple testing, although the pooled estimate was numerically larger in cross-sectional studies (OR = 1.41) than in longitudinal studies (OR = 1.25). This pattern is compatible with, but does not prove, bidirectionality: cross-sectional estimates can reflect both screen exposure preceding anxiety and anxious young people increasing or changing their screen use. Longitudinal studies better establish temporal ordering, but only three independent longitudinal studies contributed to the corresponding moderator subgroup in the primary OR analysis, limiting precision. Future studies should use repeated measures of both screen exposure and anxiety, distinguish between-person from within-person change, and account for baseline mental health to clarify whether increases in screen use predict subsequent increases in anxiety within the same individual. The study design classification was based on the temporal structure of the extracted effect estimate rather than merely the parent cohort label.

Outcome ascertainment also moderated the pooled association. Studies using symptom scales or cutoffs showed a stronger association (OR = 1.46) than studies based on diagnosis or condition status (OR = 1.20). One possible explanation is that symptom scales capture subclinical variation across the full anxiety continuum and may therefore be more sensitive to modest exposure-related differences, whereas diagnostic outcomes are less common and can be affected by help-seeking, parental recognition, access to care, and diagnostic procedures. The region analysis suggested a larger estimate in Asia/Middle East than in North America/Oceania; this difference met the Benjamini–Hochberg FDR criterion (q = 0.0479) but not the more conservative Holm criterion (adjusted *p* = 0.0718), and should therefore be treated as exploratory. Cultural context, academic demands, family norms, pandemic-related digital learning, and platform ecosystems could plausibly modify associations, but the current data do not permit firm regional conclusions.

Sex-specific evidence was too sparse for a well-powered formal moderator test. The available stratified estimates suggested a somewhat larger pooled association in females than males, but only three independent studies contributed explicit sex-stratified effects, and uncertainty was substantial. This is broadly consistent with prior reviews in which sex differences have been inconsistent and may depend on the type of digital activity, social comparison, sleep, or feedback sensitivity rather than sex alone (Kerr et al., 2025; Saleem et al., 2024). Future work should report sex- and gender-stratified dose data more consistently and examine whether differences reflect exposure patterns, developmental vulnerability, platform-specific behaviors, or measurement artifacts.

This study has several strengths. It focused specifically on anxiety rather than combining anxiety with depression or broad psychological distress; separated actual time-based exposure from problematic use constructs; retained multiple eligible effects using a three-level framework; explicitly mapped overlapping cohorts to reduce double counting; and evaluated the dose–response relationship using restricted cubic splines with cluster-robust and wild cluster inference. The conclusions were also supported by leave-one-study-out analyses, quality-restricted and categorical-only sensitivity analyses, and multiple small-study-effect diagnostics. To our knowledge, this combination of an anxiety-specific outcome, time-based digital media exposure, multilevel dependence modeling, and formal dose–response assessment has not been jointly addressed in previous syntheses.

Several limitations should also be acknowledged. First, the evidence base was observational and predominantly cross-sectional, so residual confounding and reverse causation cannot be excluded. Second, screen exposure was usually self- or parent-reported; duration estimates may be imprecise, particularly for fragmented smartphone and social media use. Third, anxiety was assessed using heterogeneous instruments and thresholds, and the significant outcome ascertainment moderator indicates that measurement choices may influence effect magnitude. Fourth, the primary pooled model focused on adjusted OR-family estimates; RR, PR, correlations, regression coefficients, and continuous symptom outcomes were preserved separately rather than indiscriminately converted, improving metric validity but reducing the number of studies contributing to any single pooled family. The broad OR synthesis nevertheless included heterogeneous study-defined exposure contrasts, including a small number of per-unit, transition/change, or mixed movement guideline contrasts; the pooled OR should therefore not be interpreted as the effect of a fixed increase in screen time. Reassuringly, an analysis restricted to conventional categorical higher-versus-lower contrasts yielded an essentially identical pooled estimate. Fifth, the dose–response analysis was based on only seven independent studies with sufficiently recoverable daily dose data, and several studies contained open-ended exposure categories requiring prespecified dose assignment. Although alternative dose assumptions and knot placements did not support nonlinearity, statistical power to detect complex shapes remains limited. Sixth, the available literature rarely distinguished educational from recreational use, active from passive engagement, or beneficial from harmful content, even though current guidance emphasizes these contextual dimensions. Seventh, some potentially relevant full-text reports could not be retrieved, and publication bias cannot be ruled out despite the absence of a consistent small study effect signal. Eighth, although the literature search and initial screening were primarily conducted by one researcher, the eligibility decisions, extracted data, and methodological quality assessments used in the final synthesis underwent independent verification by a second author; the single-researcher initial screening stage should nevertheless be considered when interpreting the review process.

The findings have practical implications, but they support nuanced guidance rather than a single universal cutoff. For clinicians, families, schools, and policymakers, greater duration of screen-based digital media use may be considered one marker associated with anxiety burden, particularly when high use co-occurs with sleep disruption, distressing social interactions, functional impairment, or displacement of offline activities. However, duration alone should not be used to label normal or pathological media use. Future research should prioritize device-logged exposure, repeated longitudinal measurement, clearer separation of recreational and educational screen use, platform- and activity-specific dose data, and analyses that test sleep, social comparison, cybervictimization, and offline activity as mediating mechanisms. Such designs would help determine whether the graded association observed here reflects a causal dose effect, differential vulnerability, reciprocal processes, or a combination of these pathways.

## 5. Conclusions

Greater screen-based digital-media use was associated with higher odds of anxiety among children and adolescents, and the association remained stable across multiple sensitivity and influence analyses, including a sensitivity analysis restricted to conventional categorical higher-versus-lower exposure contrasts. The dose–response evidence supported a graded positive association between increasing daily screen exposure and the observed odds of anxiety but did not provide robust evidence for a nonlinear threshold. Screen type and outcome ascertainment contributed to heterogeneity, while evidence for regional moderation was sensitive to the multiplicity-control procedure. These findings support a graded and context-sensitive interpretation of the observed association rather than a causal dose effect, and highlight the need for longitudinal, device-based, and mechanism-focused research before specific causal or threshold-based recommendations can be made.

## Figures and Tables

**Figure 1:**
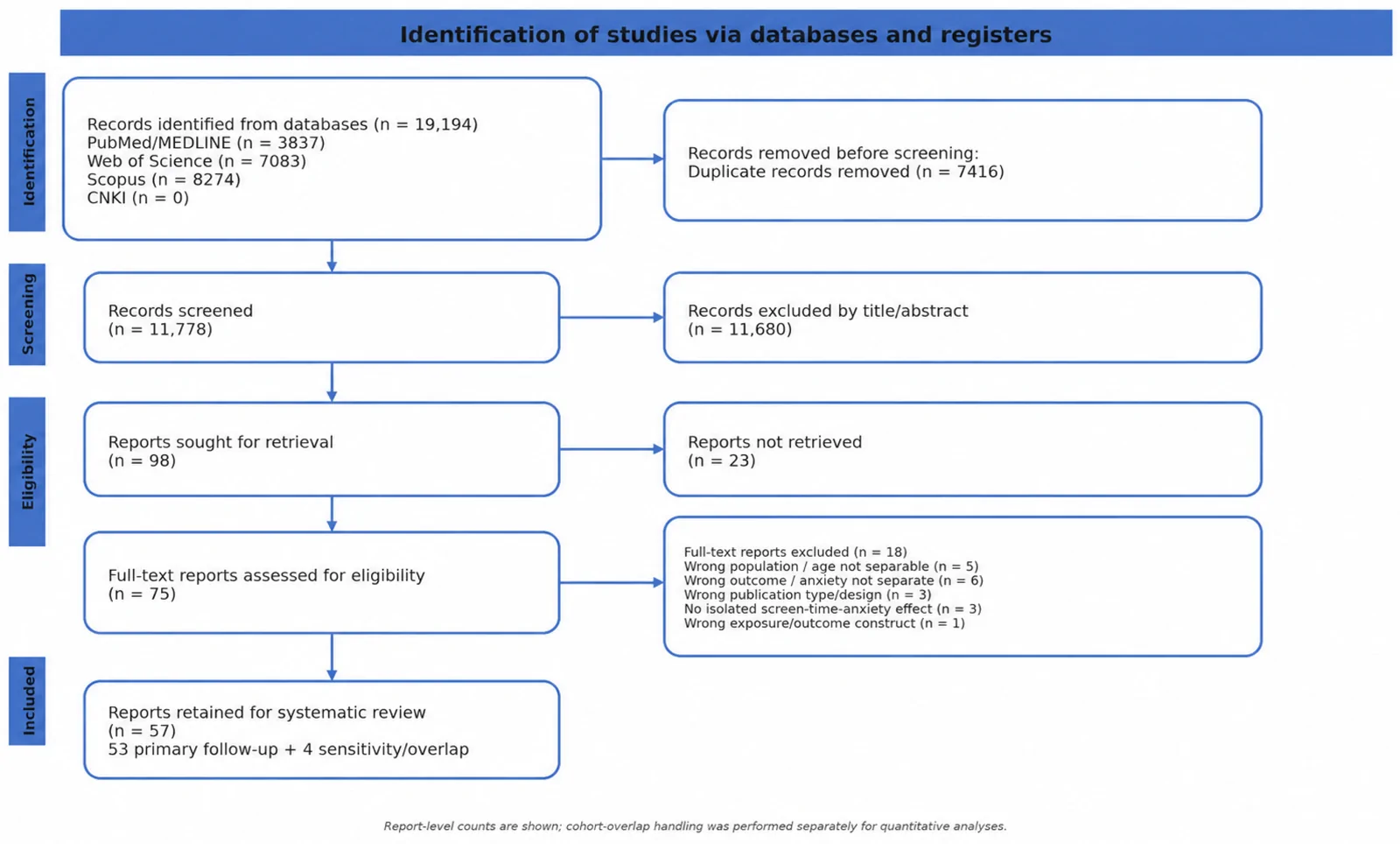
PRISMA 2020 flow diagram of study identification, screening, eligibility assessment, and inclusion. Full-text exclusion reasons are summarized in the diagram; report-specific citations and reasons are provided in Supplementary Table S4. Counts are report-level; cohort overlap handling was performed separately for quantitative analyses.

**Figure 2:**
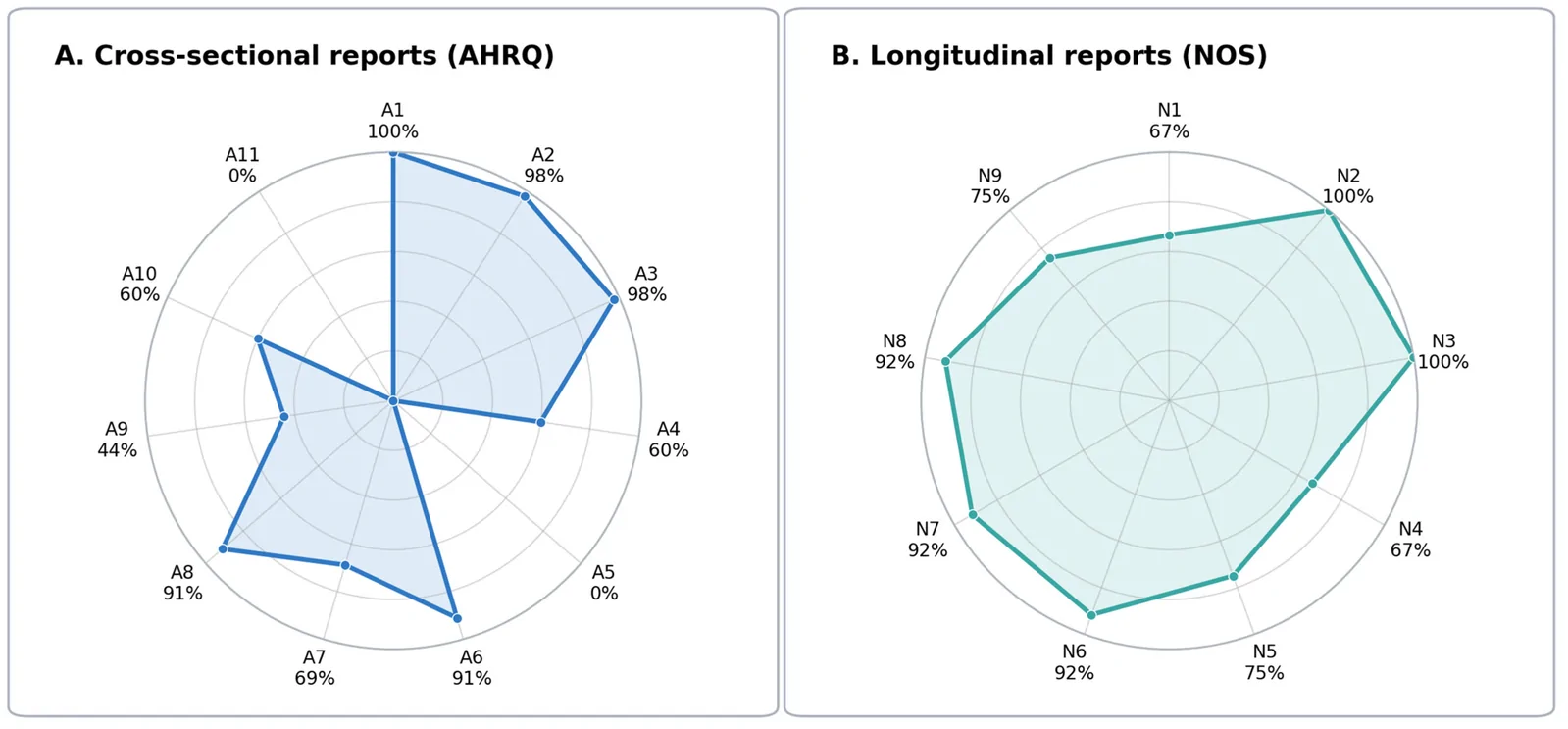
Study quality assessment of the included reports. (**A**) AHRQ item fulfillment among cross-sectional reports; (**B**) Newcastle–Ottawa Scale item fulfillment among longitudinal reports. Values represent the final consensus assessments following independent verification by a second author.

**Figure 3:**
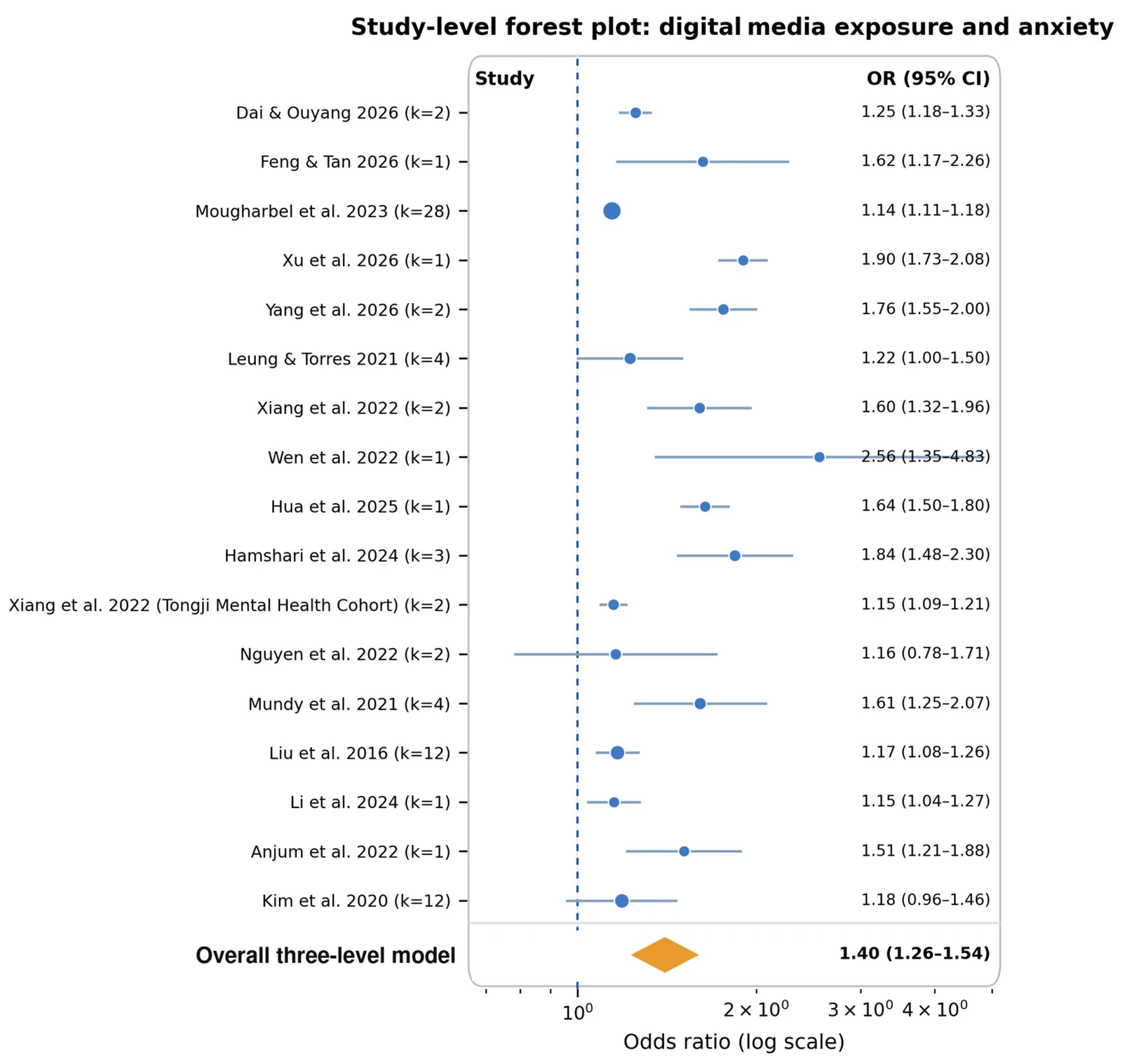
Forest plot of screen-based digital media use and anxiety. Study-level points are descriptive summaries of multiple eligible adjusted OR contrasts for visualization; the pooled diamond is the primary three-level random-effects estimate (OR = 1.40, 95% CI 1.26–1.54). The studies displayed in this figure correspond to the references listed in the reference section.

**Figure 4:**
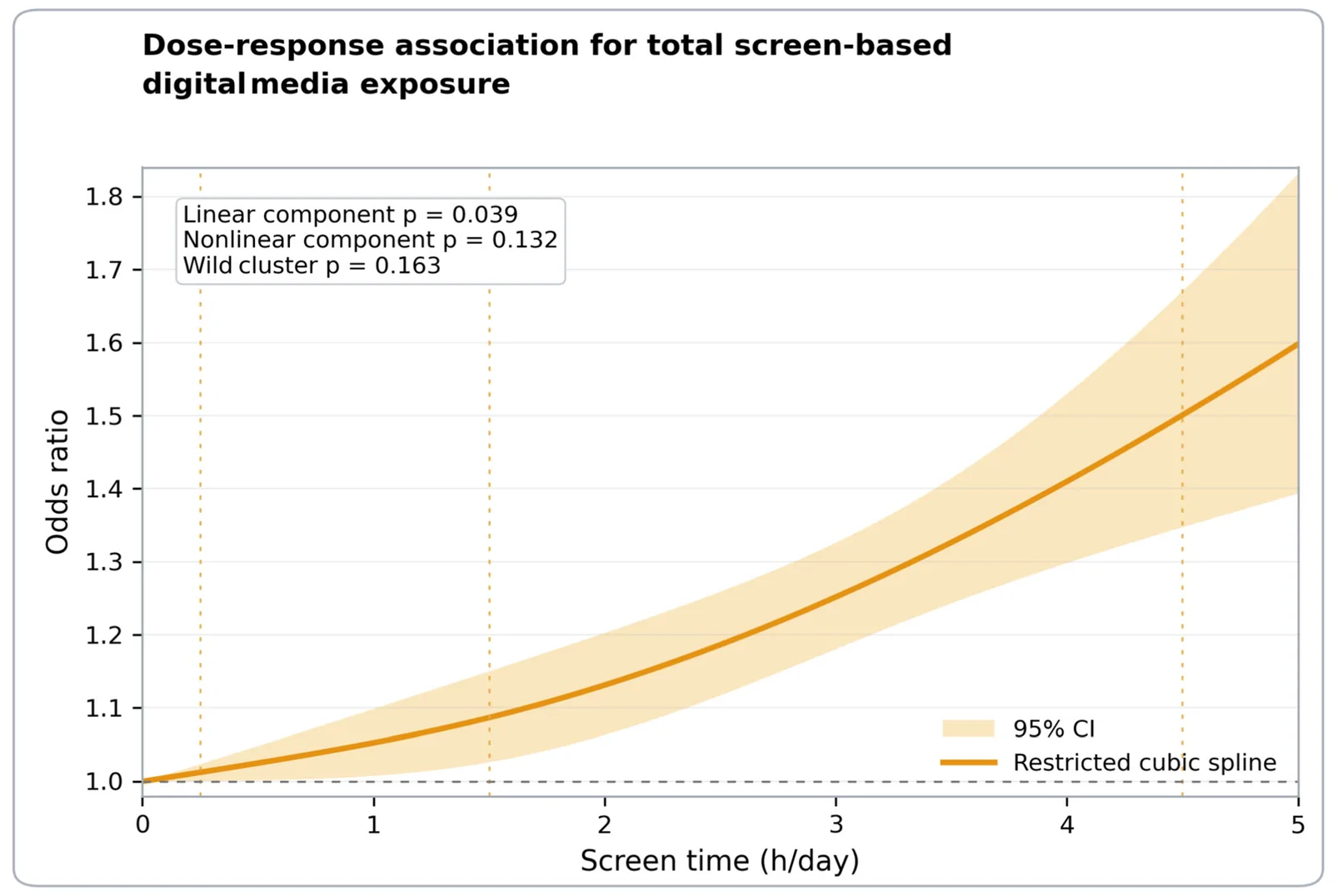
Restricted cubic spline dose–response association between daily screen-based digital media use and anxiety. The linear spline component was significant (*p* = 0.039), whereas the nonlinear component was not (cluster-robust *p* = 0.132; exact wild cluster bootstrap *p* = 0.163); therefore, no nonlinear threshold is claimed.

**Figure 5:**
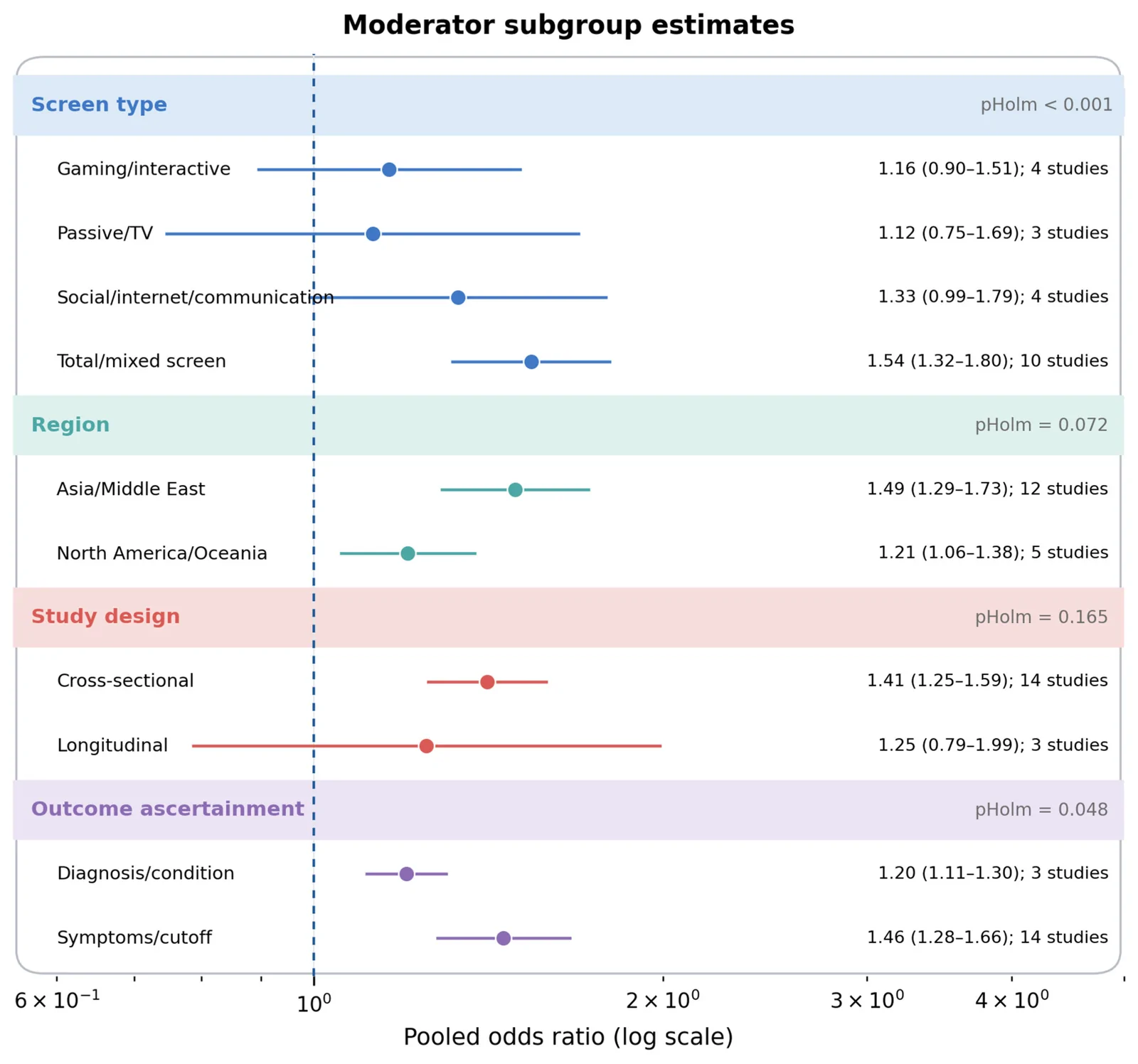
Moderator subgroup estimates for screen type, region, study design, and anxiety outcome ascertainment. Holm-adjusted *p* values were used for the primary multiplicity-controlled inference; Benjamini–Hochberg FDR results are reported as a sensitivity analysis in the text.

**Figure 6:**
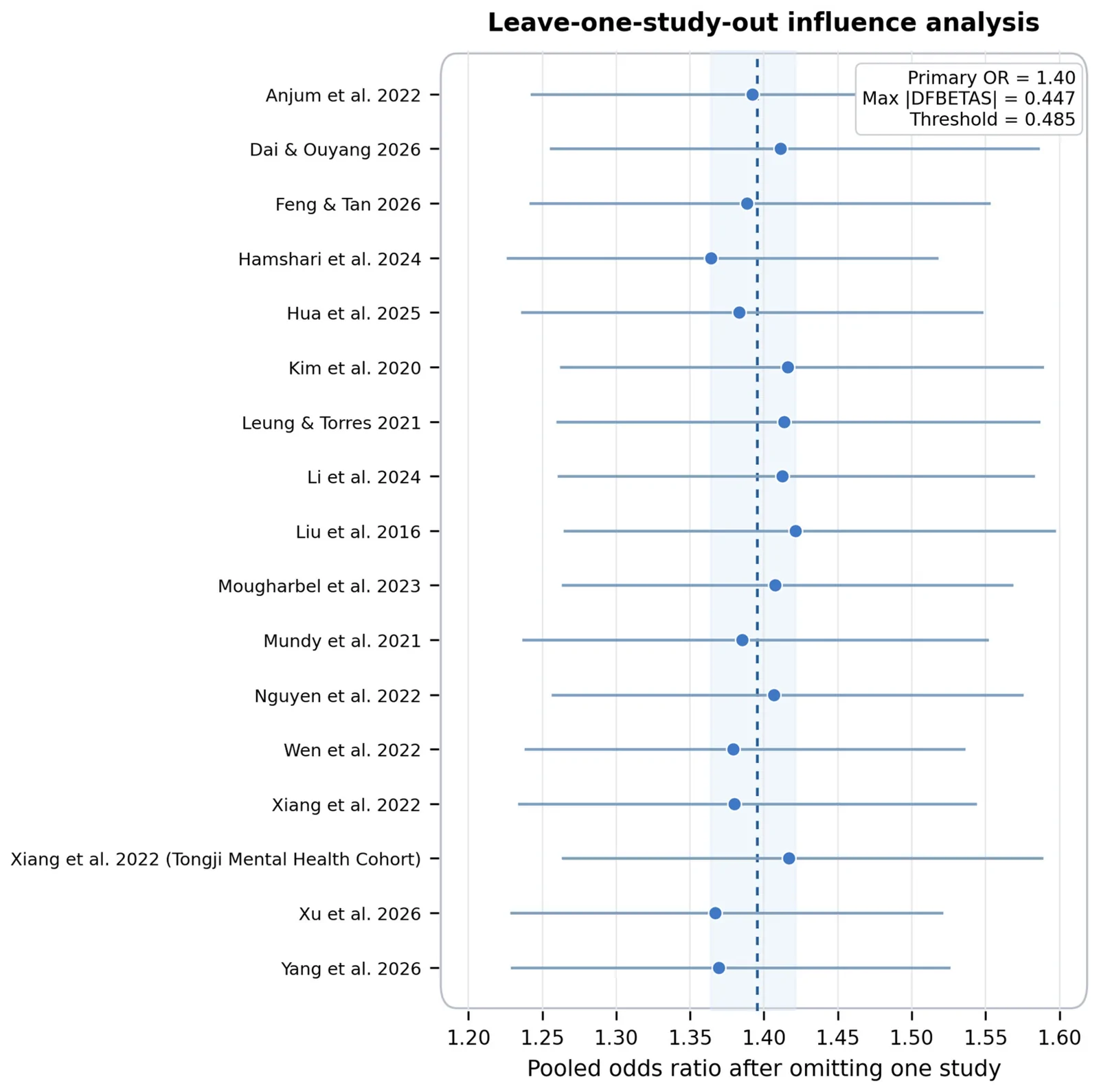
Leave-one-study-out influence analysis. The dashed vertical line denotes the primary pooled OR; no omitted study crossed the prespecified DFBETAS influence threshold. The studies displayed in this figure correspond to the references listed in the reference section.

**Figure 7:**
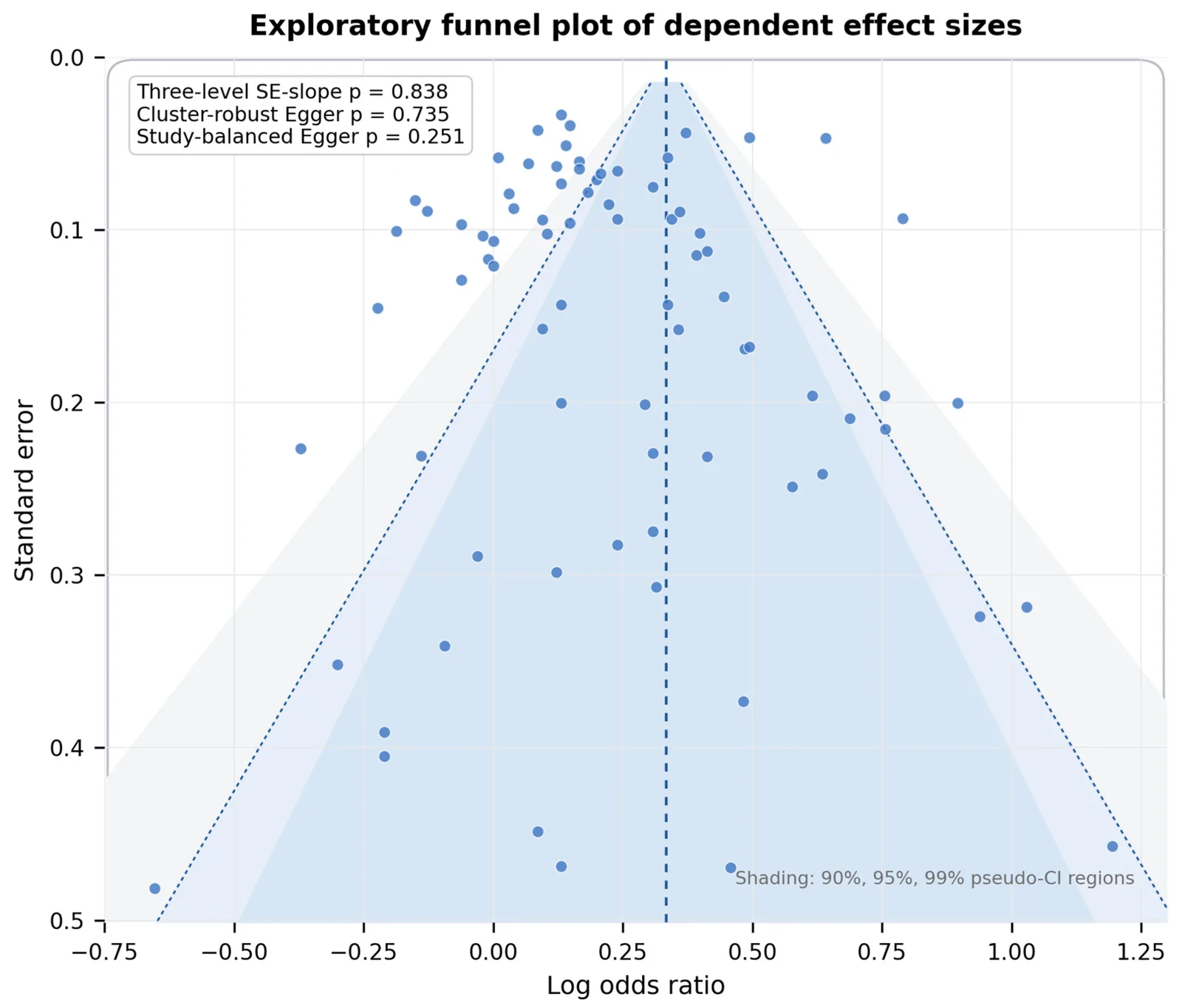
Contour-enhanced exploratory funnel plot of the 79 dependent adjusted OR-family effects. The dashed vertical line represents the pooled effect estimate, and the shaded regions indicate pseudo-confidence regions. Because multiple effects were retained per study, and contrasts differed in exposure dose and screen type, visual asymmetry was not interpreted as a standalone test of publication bias.

**Table 1:** Retained reports and analytic role.

Study	Year	Analytic Role	Dose Grade	Quality	Score
(Carlander et al., 2026)	2026	Main meta + dose	A	Moderate	7
(Yang et al., 2026)	2026	Main meta	C	Moderate	7
(Zetterqvist et al., 2026)	2026	Sensitivity	B	High	7
(Dai & Ouyang, 2026)	2026	Main meta	C	Moderate	6
(Xu et al., 2026)	2026	Main meta	C	High	8
(Feng & Tan, 2026)	2026	Main meta	B	Moderate	7
(Karakus et al., 2026)	2026	Main meta	B	High	8
(Fortier et al., 2025)	2025	Main meta	C	High	8
(Frei et al., 2025)	2025	Main meta + dose	A	High	8
(Choi et al., 2025)	2025	Main meta	C	High	9
(Sidiq et al., 2025)	2025	Main meta	B	High	8
(Nercessian & Salameh, 2025)	2025	Main meta	C	Moderate	7
(S. H. Li et al., 2025)	2025	Main meta	B	High	7
(Parida et al., 2025)	2025	Main meta	C	High	8
(Frielingsdorf et al., 2025)	2025	Main meta + dose	A	High	9
(Hua et al., 2025)	2025	Main meta	C	Moderate	7
(Zablotsky et al., 2025)	2025	Main meta	C	High	8
(Wang et al., 2025)	2025	Main meta	C	High	9
(Pedrini et al., 2024)	2024	Main meta	C	High	7
(Kosola et al., 2024)	2024	Main meta	B	High	8
(Sârbu et al., 2024)	2024	Main meta	C	Moderate	7
(Mohd Saat et al., 2024)	2024	Main meta	C	Moderate	6
(Menezes et al., 2024)	2024	Sensitivity	C	Moderate	6
(Faryabi et al., 2024)	2024	Main meta + dose	A-counts	Moderate	6
(Mandic et al., 2024)	2024	Continuous-outcome dose	A-continuous	Moderate	5
(Chen et al., 2024)	2024	Main meta + dose	A	High	9
(Francisquini et al., 2025)	2025	Main meta + dose	A	Moderate	7
(Zimmerman et al., 2024)	2024	Main meta	C	Moderate	7
(Hamshari et al., 2024)	2024	Main meta	C	Moderate	6
(Dabravolskaj et al., 2023)	2023	Main meta	C	High	7
(Menezes et al., 2023)	2023	Sensitivity	C	Moderate	6
(Mougharbel et al., 2023) COMPASS	2023	Main meta + dose	A	High	8
(Yalçınkaya et al., 2023)	2023	Continuous-outcome dose	A-continuous	Low	3
(Uddin & Hasan, 2023)	2023	Main meta + dose	A?	High	8
(Smout et al., 2023)	2023	Continuous-outcome dose	A-continuous	High	8
(D. Li et al., 2024)	2024	Main meta	B	Moderate	7
(Alsaigh et al., 2022)	2022	Main meta + dose	A-counts	Moderate	5
(Nguyen et al., 2022)	2022	Main meta + dose	A	Moderate	7
(Wen et al., 2022)	2022	Main meta	C	Moderate	6
(Z. Xiang et al., 2022) Chinese primary-school cohort	2022	Main meta	B	High	8
(Roberston et al., 2022)	2022	Main meta	C->A?	Moderate	7
(M. Xiang et al., 2022)	2022	Main meta	C	High	9
(Anjum et al., 2022)	2022	Main meta	C	High	8
(Mundy et al., 2021)	2021	Main meta + dose	A-conditional	High	9
(Leung & Torres, 2021)	2021	Main meta + dose	A	High	9
(Oliva et al., 2021)	2021	Main meta	C	Moderate	7
(Lema-Gómez et al., 2021)	2021	Main meta	B	High	8
(Boers et al., 2020)	2020	Main meta	B	Moderate	6
(Oberle et al., 2020)	2020	Main meta	C	High	8
(Kim et al., 2020)	2020	Main meta + dose	A?	High	8
(Thorisdottir et al., 2019)	2019	Main meta	B	Moderate	6
(Hrafnkelsdottir et al., 2018)	2018	Main meta	C	Moderate	6
(Yan et al., 2017)	2017	Continuous-outcome dose	A-continuous	High	8
(Liu et al., 2016)	2016	Main meta + dose	A	Moderate	6
(Gunnell et al., 2016)	2016	Sensitivity	C	High	9
(Maras et al., 2015)	2015	Main meta	C	Moderate	6
(Selfhout et al., 2009)	2009	Main meta	B	Moderate	6

**Table 2:** Main quantitative results.

Analysis	Data	Estimate	Inference
Primary three-level meta-analysis	79 effects/17 studies	OR 1.396	1.262–1.543; I^2^ total 51.7%; higher screen-based digital media use was associated with greater odds of anxiety.
Dose–response linear component	55 non-reference effects/7 studies	RCS linear component	*p* = 0.0394; evidence of an increasing dose–response trend.
Dose–response nonlinearity	55 non-reference effects/7 studies	RCS nonlinear component	*p* = 0.1316; wild cluster *p* = 0.1628; no robust evidence of nonlinearity; no threshold claimed.
Predicted OR at 2 h/day	RCS prediction	OR 1.131	95% CI 1.064–1.203; dose gradient illustration, not a clinical threshold.
Predicted OR at 4 h/day	RCS prediction	OR 1.410	95% CI 1.299–1.530; dose gradient illustration, not a clinical threshold.
Categorical higher-versus-lower sensitivity	72 effects/12 studies	OR 1.400	1.237–1.585; essentially unchanged from the broad primary estimate.
High-quality-only sensitivity	54 effects/8 studies	OR 1.355	1.175–1.563; direction and magnitude remained similar.

## Data Availability

The data supporting the reported review are included in the article and Supplementary Materials, including the search strategies, quality assessments, retained study/effect size tables, and full-text exclusion table. No public repository was established for this review. Additional data-extraction materials and analytic code are available from the corresponding author upon reasonable request.

## Supplementary Materials

The following supporting information can be downloaded at: https://www.mdpi.com/article/10.3390/bs16101774/s1, Figure S1A: effect-level forest plot of the first 40 adjusted OR-family effects; Figure S1B: effect-level forest plot of the remaining 39 effects; Supplementary Table S1: complete search strategies for PubMed/MEDLINE, Web of Science Core Collection, Scopus, and CNKI; Supplementary Table S2: AHRQ and Newcastle–Ottawa Scale quality assessments; Supplementary Table S3: retained study and effect size details; Supplementary Table S4: reports excluded after full-text assessment and reasons for exclusion; and Supplementary Methods and Sensitivity Analyses. The completed PRISMA 2020 checklist is provided separately as reporting material.
