# Supplementary material for: Dose–Response Associations Between Screen-Based Digital Media Use and Anxiety in Children and Adolescents: A Systematic Review and Three-Level Meta-Analysis
Source: Behav Sci (Basel). 2026 Sep 28;16(10):1774. doi: 10.3390/bs16101774 (PMC13701503; doi:10.3390/bs16101774)
Supplement: Supplementary file 1 [file behavsci-16-01774-s001.zip › Completed_PRISMA_revised_black.pdf]

PRISMA 2020 Checklist

Manuscript ID: behavsci-4573886

Section and Topic	Item #	Checklist item	Location where item is reported
TITLE – Title	1	Identify the report as a systematic review.	p. 1 (title)
ABSTRACT – Abstract	2	See the PRISMA 2020 for Abstracts checklist.	pp. 1–2 (Abstract; includes objective/context, eligibility, information sources, quality assessment, synthesis methods, principal results, limitations, interpretation, funding, and registration status)
INTRODUCTION – Rationale	3	Describe the rationale for the review in the context of existing knowledge.	pp. 2–4 (Introduction)
INTRODUCTION – Objectives	4	Provide an explicit statement of the objective(s) or question(s) the review addresses.	p. 4 (end of Introduction)
METHODS – Eligibility criteria	5	Specify the inclusion and exclusion criteria for the review and how studies were grouped for the syntheses.	pp. 5–6 (Section 2.3); p. 10 (Section 2.7, synthesis eligibility rules)
METHODS – Information sources	6	Specify all databases, registers, websites, organisations, reference lists and other sources searched or consulted to identify studies. Specify the date when each source was last searched or consulted.	p. 5 (Section 2.2); Supplementary Table S1 (searches conducted in September 2026)
METHODS – Search strategy	7	Present the full search strategies for all databases, registers and websites, including any filters and limits used.	p. 5 (Section 2.2); Supplementary Table S1
METHODS – Selection process	8	Specify the methods used to decide whether a study met the inclusion criteria of the review, including how many reviewers screened each record and each report retrieved, whether they worked independently, and if applicable, details of automation tools used in the process.	pp. 6–7 (Section 2.4)
METHODS – Data collection process	9	Specify the methods used to collect data from reports, including how many reviewers collected data from each report, whether they worked independently, any processes for obtaining or confirming data from study investigators, and if applicable, details of automation tools used in the process.	pp. 6–7 (Section 2.4)
METHODS – Data items	10a	List and define all outcomes for which data were sought. Specify whether all results that were compatible with each outcome domain in each study were sought, and if not, the methods used to decide which results to collect.	pp. 5–7 (Sections 2.3–2.4)
METHODS – Data items	10b	List and define all other variables for which data were sought. Describe any assumptions made about any missing or unclear information.	pp. 6–8 (Sections 2.4–2.5)
METHODS – Study risk of bias assessment	11	Specify the methods used to assess risk of bias in the included studies, including details of the tool(s) used, how many reviewers assessed each study and whether they worked independently, and if applicable, details of automation tools used in the process.	pp. 8–9 (Section 2.6); Supplementary Table S2
METHODS – Effect measures	12	Specify for each outcome the effect measure(s) used in the synthesis or presentation of results.	pp. 7–8 (Section 2.5)
METHODS – Synthesis methods	13a	Describe the processes used to decide which studies were eligible for each synthesis.	pp. 5–11 (Sections 2.3–2.7)
METHODS – Synthesis methods	13b	Describe any methods required to prepare the data for presentation or synthesis, such as handling of missing summary statistics, or data conversions.	pp. 7–8 (Section 2.5)
METHODS – Synthesis methods	13c	Describe any methods used to tabulate or visually display results of individual studies and syntheses.	pp. 10–11 (Section 2.7)
METHODS – Synthesis methods	13d	Describe any methods used to synthesize results and provide a rationale for the choice(s). If meta-analysis was performed, describe the model(s), methods to identify the presence and extent of statistical heterogeneity, and software package(s) used.	pp. 9–10 (Section 2.7)
METHODS – Synthesis methods	13e	Describe any methods used to explore possible causes of heterogeneity among study results.	p. 10 (Section 2.7)
METHODS – Synthesis methods	13f	Describe any sensitivity analyses conducted to assess robustness of the synthesized results.	pp. 10–11 (Section 2.7); Supplementary Methods and Sensitivity Analyses
METHODS – Reporting bias assessment	14	Describe any methods used to assess risk of bias due to missing results in a synthesis (arising from reporting biases).	p. 10 (Section 2.7)
METHODS – Certainty assessment	15	Describe any methods used to assess certainty (or confidence) in the body of evidence for an outcome.	p. 10 (Section 2.7: no formal certainty-of-evidence framework was applied)

Section and Topic	Item #	Checklist item	Location where item is reported
RESULTS – Study selection	16a	Describe the results of the search and selection process, from the number of records identified in the search to the number of studies included in the review, ideally using a flow diagram.	pp. 11–12 (Section 3.1 and Figure 1)
RESULTS – Study selection	16b	Cite studies that might appear to meet the inclusion criteria, but which were excluded, and explain why they were excluded.	pp. 11–12 (Section 3.1 and Figure 1); Supplementary Table S4
RESULTS – Study characteristics	17	Cite each included study and present its characteristics.	pp. 26–28 (Table 1); Supplementary Table S3; References pp. 29–38
RESULTS – Risk of bias in studies	18	Present assessments of risk of bias for each included study.	pp. 12–13 (Section 3.2 and Figure 2); Supplementary Table S2
RESULTS – Results of individual studies	19	For all outcomes, present, for each study: (a) summary statistics for each group where appropriate and (b) an effect estimate and its precision, ideally using structured tables or plots.	Supplementary Table S3; Figures S1A–S1B
RESULTS – Results of syntheses	20a	For each synthesis, briefly summarise the characteristics and risk of bias among contributing studies.	pp. 11–13 (Sections 3.1–3.3); pp. 26–28 (Table 1); Supplementary Table S2
RESULTS – Results of syntheses	20b	Present results of all statistical syntheses conducted. If meta-analysis was done, present for each the summary estimate and its precision and measures of statistical heterogeneity. If comparing groups, describe the direction of the effect.	pp. 13–20 (Sections 3.3–3.6); p. 28 (Table 2)
RESULTS – Results of syntheses	20c	Present results of all investigations of possible causes of heterogeneity among study results.	pp. 16–17 (Section 3.5 and Figure 5)
RESULTS – Results of syntheses	20d	Present results of all sensitivity analyses conducted to assess the robustness of the synthesized results.	pp. 13, 15–20; p. 28 (Table 2); Supplementary Methods and Sensitivity Analyses
RESULTS – Reporting biases	21	Present assessments of risk of bias due to missing results (arising from reporting biases) for each synthesis assessed.	pp. 17–20 (Section 3.6 and Figure 7)
RESULTS – Certainty of evidence	22	Present assessments of certainty (or confidence) in the body of evidence for each outcome assessed.	pp. 10 and 20 (no formal GRADE-style certainty rating was assigned; explicitly stated)
DISCUSSION – Discussion	23a	Provide a general interpretation of the results in the context of other evidence.	pp. 21–25 (Discussion)
DISCUSSION – Discussion	23b	Discuss any limitations of the evidence included in the review.	pp. 24–25 (Discussion)
DISCUSSION – Discussion	23c	Discuss any limitations of the review processes used.	p. 25 (Discussion)
DISCUSSION – Discussion	23d	Discuss implications of the results for practice, policy, and future research.	p. 25 (Discussion and Conclusions)
OTHER INFORMATION – Registration and protocol	24a	Provide registration information for the review, including register name and registration number, or state that the review was not registered.	p. 4 (Section 2.1: review was not prospectively registered)
OTHER INFORMATION – Registration and protocol	24b	Indicate where the review protocol can be accessed, or state that a protocol was not prepared.	p. 4 (Section 2.1: no formal review protocol was prepared)
OTHER INFORMATION – Registration and protocol	24c	Describe and explain any amendments to information provided at registration or in the protocol.	p. 4 (Section 2.1: not applicable because the review was not registered and no formal protocol was prepared)
OTHER INFORMATION – Support	25	Describe sources of financial or non-financial support for the review, and the role of the funders or sponsors in the review.	p. 29 (Funding)
OTHER INFORMATION – Competing interests	26	Declare any competing interests of review authors.	p. 29 (Conflicts of Interest)
OTHER INFORMATION – Availability of data, code and other materials	27	Report which review materials are publicly available and where they can be found, including data extraction forms/data, data used for analyses, analytic code, and other materials.	p. 29 (Data Availability Statement); Supplementary Tables S1–S4 and Supplementary Methods

Source: Page MJ, McKenzie JE, Bossuyt PM, Boutron I, Hoffmann TC, Mulrow CD, et al. The PRISMA 2020 statement: an updated guideline for reporting systematic reviews. BMJ. 2021;372:n71. Checklist wording reproduced under CC BY 4.0. Page locations refer to the revised manuscript rendered for resubmission.
