# Supplementary material for: Dose–Response Associations Between Screen-Based Digital Media Use and Anxiety in Children and Adolescents: A Systematic Review and Three-Level Meta-Analysis
Source: Behav Sci (Basel). 2026 Sep 28;16(10):1774. doi: 10.3390/bs16101774 (PMC13701503; doi:10.3390/bs16101774)
Supplement: Supplementary file 1 [file behavsci-16-01774-s001.zip › Supplementary_Methods_and_Sensitivity_Analyses_revised_red.pdf]

Supplementary Methods and Sensitivity Analyses

Dose–Response Associations Between Screen-Based Digital Media Use and Anxiety in Children and Adolescents

S1. Effect-Size Harmonization

Adjusted odds ratios (ORs) were the primary effect measure for the main quantitative synthesis. Effect directions were harmonized so that OR > 1 represented greater anxiety with greater screen-based digital-media exposure. ORs were analyzed on the natural-logarithmic scale because their sampling distribution is generally more nearly normal and confidence intervals more symmetric on that scale, while multiplicative associations become additive for inverse-variance modeling; pooled estimates were exponentiated back to the OR scale for presentation. For 95% confidence intervals, the standard error of the log OR was calculated as SE = [ln(upper CI) − ln(lower CI)]/(2 × 1.96); 99% confidence intervals used 2.576. Risk ratios, prevalence ratios, and hazard ratios were not automatically relabeled as ORs. Crude reconstructed ORs and non-OR metrics were reserved for secondary or sensitivity analyses.

Multiple eligible effects within a study (e.g., sex strata, screen types, anxiety subtypes, exposure categories, or assessment occasions) were retained rather than reduced to a single contrast. The primary model therefore used a three-level structure. Level 1 was the effect-specific sampling variance ($v_i = SE_i^2$), treated as an input to the model; Level 2 represented residual heterogeneity among effects within a study; and Level 3 represented heterogeneity between independent studies.

S2. Three-Level Meta-Analysis

The primary adjusted OR-family model included 79 effects from 17 independent studies. A three-level random-effects model was estimated with restricted maximum likelihood. The pooled OR was 1.396 (95% CI 1.262–1.543), with total I^2 = 51.7% (approximately 25.8% within-study and 25.9% between-study heterogeneity). The 95% prediction interval was 0.910–2.139.

To assess contrast-scale heterogeneity, an additional reviewer-requested sensitivity analysis excluded seven per-unit, transition/change, or mixed movement-guideline effects and retained 72 conventional categorical higher-versus-lower effects from 12 independent studies. The pooled OR was 1.400 (95% CI 1.237–1.585), essentially unchanged from the broad primary estimate.

Model	Effects	Studies	Pooled OR	95% CI	I^2 total	Prediction interval
Primary adjusted OR-family	79	17	1.396	1.262–1.543	51.7%	0.910–2.139
Sensitivity: all available OR-family	80	18	1.374	1.244–1.518	52.7%	0.890–2.121
Sensitivity: strict conventional contrasts	73	13	1.379	1.227–1.550	52.3%	0.885–2.148
Sensitivity: categorical higher-vs-lower only	72	12	1.400	1.237–1.585	53.0%	0.890–2.202

S3. Dose–Response Modelling

The primary dose-response analysis was restricted to exposure expressed in hours/day. Fifty-five non-reference effects representing 19 dose curves from 7 independent studies were included. A three-knot restricted cubic spline used knots at the 10th, 50th, and 90th percentiles of assigned doses (0.25, 1.50, and 4.50 h/day). "Restricted" indicates that the piecewise cubic function is constrained to be linear beyond the boundary knots, limiting unstable curvature in the tails while retaining nonlinear flexibility within the observed range. Each non-reference category was coded as the difference between the spline basis at the category dose and the spline basis at the published reference dose.

Inverse-variance weighting was combined with generalized method-of-moments residual heterogeneity. The independent study was the clustering unit. A study-cluster sandwich covariance estimator was used to account approximately for dependence among multiple curves/effects and shared reference groups. Because only seven independent study clusters contributed to the primary spline model, inference used study-cluster degrees of freedom and was supplemented by exact enumeration of all 2^7 = 128 study-level Rademacher sign patterns under the restricted linear null.

The linear spline component was statistically significant (p = 0.0394), whereas the nonlinear component was not (cluster-robust p = 0.1316; exact wild-cluster p = 0.1628). Accordingly, no nonlinear threshold or change point was claimed.

Sensitivity model	Effects	Studies	Knots	Linear p	Nonlinear p	OR at 4 h/day	95% CI at 4 h/day
Primary h/day, adjacent-width	55	7	0.25, 1.50, 4.50	0.0394	0.1316	1.410	1.299–1.530
Include h/school-day	67	8	0.25, 1.50, 3.60	0.1058	0.6734	1.437	1.268–1.628
Open upper midpoint=1.2×lower	55	7	0.25, 1.50, 4.80	0.0477	0.0930	1.408	1.296–1.528
Open upper midpoint=1.5×lower	55	7	0.25, 1.50, 6.00	0.0091	0.2809	1.308	1.222–1.400
Knots 5/50/95	55	7	0.25, 1.50, 5.00	0.0298	0.1246	1.409	1.299–1.529
Knots 25/50/75	55	7	0.75, 1.50, 3.00	0.0501	0.1657	1.409	1.295–1.533

S3.1. Leave-One-Study-Out Dose-Response Sensitivity

Leave-one-study-out spline analyses were used to assess the stability of the nonlinear component. Six of seven omissions yielded non-significant nonlinearity; omitting Mundy et al. 2021 yielded p = 0.0379. Because the overall nonlinear test and wild-cluster test were non-significant and the leave-one-study-out pattern was not uniformly stable, a nonlinear threshold was not reported.

Excluded study	Studies remaining	Effects remaining	Nonlinear p	OR at 4 h/day
Dai & Ouyang 2026	6	53	0.2295	1.435
Hamshari et al. 2024	6	52	0.1294	1.380
Kim et al. 2020	6	43	0.1895	1.415
Leung & Torres 2021	6	51	0.1901	1.396
Mougharbel et al. 2023	6	27	0.6909	1.661
Mundy et al. 2021	6	51	0.0379	1.407
Nguyen et al. 2022	6	53	0.1522	1.414

S4. Moderator Analyses

Prespecified moderators were screen type, region, study design, and anxiety-outcome ascertainment. Three-level meta-regression retained the nested effect structure. The independent study was treated as the clustering unit, and cluster-robust standard errors were obtained from a study-level sandwich covariance estimator applied to the fitted three-level model with CR1-type finite-sample scaling, $[G/(G-1)]\times[(n-1)/(n-p)]$. Omnibus moderator tests used F statistics with numerator degrees of freedom equal to the number of tested moderator coefficients and denominator degrees of freedom $G-p$. Four omnibus tests were adjusted with the Holm procedure as the primary multiplicity control and, as a sensitivity analysis, with the Benjamini–Hochberg false discovery rate procedure. Sex-stratified results were descriptive because only three independent studies provided explicit male/female strata.

Moderator	Effects	Studies	F	Raw p	Holm-adjusted p	BH-FDR q	Interpretation
Screen type	79	17	24.454	0.00001	0.00005	0.00005	Significant (Holm and BH-FDR)
Region	79	17	5.311	0.03590	0.07180	0.04787	BH-FDR only; exploratory
Study design	79	17	2.126	0.16545	0.16545	0.16545	Not significant
Outcome ascertainment	79	17	7.350	0.01609	0.04828	0.03219	Significant (Holm and BH-FDR)

S5. Influence and Small-Study Effects

Leave-one-study-out analyses were conducted for the primary adjusted OR model. The prespecified influence criterion was |DFBETAS| > 2/√17 = 0.485. No study exceeded this threshold; the maximum absolute DFBETAS was 0.447. Pooled ORs after omitting one study ranged from approximately 1.36 to 1.42.

Excluded study	Effects removed	Pooled OR	95% CI	|DFBETAS|
Anjum et al. 2022	1	1.392	1.243–1.559	0.049
Dai & Ouyang 2026	2	1.411	1.256–1.585	0.218
Feng & Tan 2026	1	1.389	1.242–1.552	0.098
Hamshari et al. 2024	3	1.364	1.227–1.517	0.447
Hua et al. 2025	1	1.383	1.236–1.547	0.173
Kim et al. 2020	12	1.416	1.263–1.588	0.288
Leung & Torres 2021	4	1.414	1.260–1.586	0.255
Li et al. 2024	1	1.413	1.261–1.582	0.238
Liu et al. 2016	12	1.421	1.265–1.597	0.359
Mougharbel et al. 2023	28	1.408	1.264–1.568	0.169
Mundy et al. 2021	4	1.385	1.237–1.551	0.145
Nguyen et al. 2022	2	1.407	1.257–1.574	0.159
Wen et al. 2022	1	1.379	1.239–1.535	0.233
Xiang et al. 2022	2	1.380	1.235–1.543	0.218
Xiang et al. 2022 (Tongji Mental Health Cohort)	2	1.417	1.264–1.588	0.297
Xu et al. 2026	1	1.367	1.229–1.520	0.405
Yang et al. 2026	2	1.369	1.229–1.525	0.371

Small-study effects were evaluated with a three-level standard-error meta-regression and cluster-robust Egger-type diagnostics. The funnel plot was treated as exploratory because effect sizes were dependent and represented heterogeneous exposure contrasts.

Diagnostic	Estimate/test	p value	Interpretation
Three-level small-study meta-regression: log(OR) ~ SE	SE slope = 0.1162	0.838	No evidence that effect magnitude increases with sampling SE. Preferred small-study-effect test because it retains the multilevel structure.
Cluster-robust Egger (all dependent effects)	Intercept = 0.1636	0.735	No significant funnel asymmetry by the standardized Egger intercept.
Study-balanced cluster-robust Egger	Intercept = 0.9157	0.251	No significant funnel asymmetry after giving each study equal total weight.
Funnel plot	Exploratory only		Effects are dependent and contrasts differ in dose/screen type; visual asymmetry is not proof of publication bias.
Overall conclusion	No consistent statistical evidence of small-study effects		Publication bias cannot be excluded, but multilevel/cluster-robust diagnostics do not show a consistent small-study signal.

S6. Study-Quality Sensitivity

Cross-sectional reports were assessed with the 11-item AHRQ checklist and longitudinal reports with the Newcastle–Ottawa Scale. The final consensus ratings included 29 high-quality, 27 moderate-quality, and 1 low-quality report. Restricting the primary model to high-quality studies yielded a pooled OR of 1.355 (95% CI 1.175–1.563), supporting the stability of the direction and approximate magnitude of the main association.

Model	Effects	Studies	Pooled OR	95% CI	Interpretation
Primary adjusted OR model	79	17	1.396	1.262–1.543	All quality levels in the primary adjusted OR set.
Moderate-or-high quality only	79	17	1.396	1.262–1.543	Excludes low-quality studies only.
High-quality studies only	54	8	1.355	1.175–1.563	Restrictive quality sensitivity; lower power and narrower study base.

S7. Interpretation Rules

The broad pooled OR represents an average association across heterogeneous study-defined screen-exposure contrasts; it is not the OR for one fixed number of hours/day. Time-specific interpretation is therefore based on the dose-response model. A categorical-only sensitivity analysis excluding seven per-unit, transition/change, or mixed movement-guideline effects yielded an essentially unchanged pooled OR (1.400, 95% CI 1.237–1.585), supporting robustness to contrast-scale heterogeneity. Moderator findings are exploratory and do not establish causal effect modification. Regional moderation met the Benjamini–Hochberg FDR criterion but not the more conservative Holm criterion and was therefore interpreted cautiously. No clinical or behavioral threshold is inferred because robust statistical evidence of nonlinearity was not observed.
